# Susceptibility of various Gram-negative bacteria to antibacterial agents: SMART in China 2019–2020

**DOI:** 10.1186/s12866-024-03526-8

**Published:** 2024-12-19

**Authors:** Ying Fu, Yueliang Chen, Yanfei Wang, Bingyan Yao, Pengcheng Li, Yunsong Yu

**Affiliations:** 1 Department of Clinical Laboratory, Sir Run Run Shaw Hospital, School of Medicine, Zhejiang University, Hangzhou, 310016 China; 2 Key Laboratory of Precision Medicine in Diagnosis and Monitoring Research of Zhejiang Province, Hangzhou, 310016 China; 3 Department of Infectious Diseases, Sir Run Run Shaw Hospital, School of Medicine, Zhejiang University, Hangzhou, 310016 China; 4 Key Laboratory of Microbial Technology and Bioinformatics of Zhejiang Province, Hangzhou, 310012 China; 5 V&I, Global Medical & Scientific Affairs, MSD China, Shanghai, 200233 China

**Keywords:** SMART, *Enterobacteriaceae*, ESBL, Carbapenem-resistant Enterobacterales, DTR *P. Aeruginosa*

## Abstract

**Background:**

The Study for Monitoring Antimicrobial Resistance Trends (SMART) is an international surveillance program longitudinally monitoring aerobic and facultative Gram-negative bacteria (GNB) involvement in infections and their antimicrobial resistance profiles. Here the incidence and resistance patterns of Chinese GNB isolates from bloodstream infections (BSI), intraabdominal infections (IAI), respiratory tract infections (RTI) and urinary tract infections (UTI) to commonly used antibacterial agents has been updated. 4,975 GNB isolates collected from 22 hospitals across 7 regions of China from 2019 to 2020 were analyzed. Antimicrobial minimum inhibitory concentrations were assessed using broth microdilution, and susceptibility interpretations followed the breakpoints of European Committee on Antimicrobial Susceptibility Testing 2022 or Clinical and Laboratory Standards Institute.

**Results:**

This study affirmed that *Escherichia coli* (Ec) was the most commonly identified GNB (32.1%) and then *Klebsiella pneumoniae* (Kp) (25.3%), *Pseudomonas aeruginosa* (Pa) (13.9%) and *Acinetobacter baumannii* (10.5%). The detection rates of carbapenem-resistant (CR) Enterobacterales varied across major infection sites, ranging from 10.3% in UTI to 18.9% in RTI. Specifically, the detection rates of CR-Kp and CR-Pa ranged from 16.2% in IAI to 35.8% in UTI and from 16.1% in UTI to 38.0% in RTI, respectively. Extended-spectrum β-lactamases (ESBL)-producing Ec and Kp bacteria exhibited over 91.7% susceptibility to carbapenems and at least 87.8% susceptibility to amikacin and colistin, but showed lower susceptibility to piperacillin/tazobactam (57.5–86.2%), levofloxacin (10.8–39.7%) and aztreonam (15.3–27.6%) across different infection sources. Amikacin showed higher efficacy against CR strains compared to other commonly used antibacterial drugs, with 80.0% susceptibility against CR-Ec and 82.3% susceptibility against CR-Pa, while only 36.3% susceptibility was observed against CR-Kp.

**Conclusions:**

The study found varying incidences of CR isolates in Chinese hospitals. Treatment options remained limited due to resistance to multiple antibacterial agents. Carbapenems demonstrated effective antimicrobial in vitro activity against ESBL-producing Enterobacterales found in BSI, IAI, UTI and RTI, outperforming broad-spectrum cephalosporins and other β-lactamase inhibitors.

**Supplementary Information:**

The online version contains supplementary material available at 10.1186/s12866-024-03526-8.

## Background

 Antimicrobial resistance (AMR) is a significant threat to health, reducing the effectiveness of antibiotics against bacterial infections and a study on AMR’s global burden in 2019 reported that circa 4.95 million died because of bacterial AMR. There were essential differences in world regions with western sub-Saharan Africa having the highest (27.3 per 100,000) and Australasia the lowest (6.5 per 100,000) all-age death rates attributable to AMR. Studies have revealed substantial data gaps, especially in low-income countries, highlighting the requirement for expanded microbiology laboratory facilities and data collection to better understand and address AMR [1].

Research on 369 diseases and injuries in 204 countries and territories noted that 6 infections were the top 10 causes of disability-adjusted life-years in children aged < 10 years in 2019 [2]. A 2019 study on 33 bacterial pathogens found that millions of deaths were attributable to 33 pathogens, ranking them the second leading cause of death globally in that year. Also, in that study, the burden of bacterial infections varied significantly by region, with lower-income countries experiencing higher mortality rates due to limited access to healthcare and antibiotics [3]. The studies indicated that continued research and data collection would be essential to monitor trends in bacterial infections. The Global Antimicrobial Resistance and Use Surveillance System (GLASS) is a pivotal initiative launched by the World Health Organization (WHO) to combat AMR and represents a comprehensive approach to AMR surveillance, emphasizing standardized data collection and analysis, capacity building, and collaborative efforts. By integrating diverse data sources and focusing on both human and environmental aspects of AMR, GLASS aims to inform and enhance strategies to combat AMR effectively [4]. In addition, the WHO concomitantly published a Bacterial Priority Pathogens List (BPPL), in which antibiotic-resistant “priority pathogens” posing the greatest threat to the health of human were listed. The WHO emphasized that especially Gram-negative bacteria (GNB) can pass genetic material related to drug-resistance to other bacteria [5] and the emergence of AMR among GNB poses a formidable hurdle in combating infectious diseases [6, 7], since horizontal gene transfer can cause rapid changes in bacterial resistance to antimicrobial drugs [8]. Of all the antibacterial agents available to humans, carbapenems such as imipenem (IPM), doripenem and ertapenem (ETP) stand as the antibacterial agents of last resort, often reserved for infections resistant to commonly-used antibacterial agents [9]. However, instances of carbapenem-resistant infections, particularly those caused by the Enterobacteriaceae and other GNB, have surfaced, raising valid concerns that we may have reached the end of the pipeline of antibacterial agents [10, 11]. To address these challenges, epidemiological surveillance has been implemented to monitor resistance trends to guide initial empirical selection of antibacterial agents for the treatment of infections.

The Study for Monitoring Antimicrobial Resistance Trends (SMART) has been an international program longitudinally monitoring aerobic and facultative GNB involvement in infections and their AMR profiles [12–14]. The present study delves into surveillance data from 2019 to 2020, focusing on the susceptibility of GNB isolated from urinary tract infections (UTI), bloodstream infections (BSI), intraabdominal infections (IAI) and respiratory tract infections (RTI) in China, to provide an updated perspective on the ongoing SMART program.

## Methods

There were 18 hospitals enrolled in the study in 2019 and 4 hospitals in 2020. Each year hospitals in the SMART program were asked to consecutively collect Gram-negative isolates, including up to 50 isolates for BSI, IAI and UTI, and up to 100 isolates for RTI. Minimum inhibitory concentrations (MICs) of antibacterial agents were evaluated using the microdilution method on a Trek Diagnostic System and susceptibility interpretations were based on the breakpoints of Clinical and Laboratory Standards Institute (CLSI) M100, 32nd edition [15], with the exception of colistin (COL). The COL susceptibility testing results were interpreted by applying 2022 European Committee on Antimicrobial Susceptibility Testing (EUCAST) breakpoints [16]. Extended-spectrum β-lactamases-producing Enterobacterales (ESBL-E) were defined as species of Enterobacterales that were non-susceptible to ceftriaxone (CRO) if the MIC was ≥ 2 µg/mL but susceptible to carbapenems. Non-ESBL-E were defined as being susceptible to CRO. Carbapenem resistance (CR) was defined as Gram-negative organisms being resistant to ETP, IPM or meropenem (MEM). For *Proteus spp.*, CR was defined as resistance to ETP or MEM, and for *Pseudomonas aeruginosa* (Pa), CR was defined as resistance to IPM or MEM. Difficult-to-treat resistance (DTR) Pa isolates are non-susceptible to all of the following compounds: IPM, MEM, ceftazidime (CAZ), cefepime (FEP), levofloxacin (LVX), aztreonam (ATM) and piperacillin/tazobactam (TZP) [17].

The Ethics Committee of Sir Run Run Shaw Hospital, Zhejiang University School of Medicine, approved the study protocol (Approval No: 20210811-33) and agreed to waive patient informed consent due to the retrospective nature of the study and because the specimens were obtained from previous treatments and diagnoses for patients.

### Statistical analysis

Statistical analyses of antimicrobial susceptibility were conducted using SAS ver. 14 (Cary, NC, USA).

## Results

### Distribution of GNB infections in different infection sites of patients from 2019 to 2020

A total of 4,956 strains of GNB from 2019 to 2020 were collected for BSI (984, 19.9%), UTI (991, 20.0%), RTI (1,990, 40.2%) and IAI (991, 20.0%). The top three pathogens for BSI, UTI and IAI were *Escherichia coli* (Ec), *Klebsiella pneumoniae* (Kp) and *Pa*, respectively while the top three infectious pathogens for RTI were Kp, Pa and Ec (Table 1).

**Table 1:** Distribution of the most frequent Gram-negative bacteria isolated from different infection sites

Microorganisms	Total	BSI	UTI	RTI	IAI
Total	4,956	984	991	1,990	991
*Escherichia coli*	1,589 (32.1)	446 (45.3)	574 (57.9)	146 (7.3)	423 (42.7)
*Klebsiella pneumoniae*	1,254 (25.3)	268 (27.2)	176 (17.8)	575 (28.9)	235 (23.7)
*Pseudomonas aeruginosa*	688 (13.9)	63 (6.4)	56 (5.7)	497 (25.0)	72 (7.3)
*Acinetobacter baumannii*	522 (10.5)	43 (4.4)	37 (3.7)	392 (19.7)	50 (5.1)
*Stenotrophomonas maltophilia*	130 (2.6)	9 (0.9)	6 (0.6)	96 (4.8)	19 (1.9)
*Enterobacter cloacae*	120 (2.4)	32 (3.3)	17 (1.7)	35 (1.8)	36 (3.6)
*Klebsiella aerogenes*	88 (1.8)	12 (1.2)	10 (1.0)	45 (2.3)	21 (2.1)
*Proteus mirabilis*	79 (1.6)	8 (0.8)	37 (3.7)	17 (0.9)	17 (1.7)
*Klebsiella oxytoca*	68 (1.4)	22 (2.2)	9 (0.9)	21 (1.1)	16 (1.6)
*Serratia marcescens*	52 (1.1)	10 (1.0)	8 (0.8)	31 (1.6)	3 (0.3)
*Acinetobacter spp.*	38 (0.8)	6 (0.6)	5 (0.5)	23 (1.2)	4 (0.4)
*Citrobacter freundii*	38 (0.8)	7 (0.7)	13 (1.3)	7 (0.4)	11 (1.1)
*Klebsiella spp.*	29 (0.6)	4 (0.4)	0 (0.0)	18 (0.9)	7 (0.7)
*Enterobacter spp.*	29 (0.6)	5 (0.5)	5 (0.5)	11 (0.6)	8 (0.8)
*Acinetobacter pittii*	26 (0.5)	0 (0.0)	5 (0.5)	16 (0.8)	5 (0.5)
*Enterobacter hormaechi*	26 (0.5)	4 (0.4)	2 (0.2)	7 (0.4)	13 (1.3)
Others	180 (3.6)	45 (4.6)	31 (3.1)	53 (2.7)	51 (5.1)

Data are presented as numbers (percentages)

### Susceptibility of GNB to conventional antibacterial agents from 2019 to 2020

The susceptibility of GNB to conventional antibacterial agents varied. Among the pathogens, the susceptibilities of Ec to CAZ, CRO, FEP, ATM and LVX were lower than 60%. For TZP and cefoxitin (FOX) the susceptibilities were 74.7 – 85.2%, and to MEM, IPM, ETP, amikacin (AMK) and COL the susceptibilities were all over 90%. The susceptibility of Kp to COL was 94.3% and to carbapenems (IPM, ETP, MEM) was 73.5 – 76.0% and to AMK 82.7%; for other antibacterial agents, susceptibilities were < 63.6%. Pa was resistant to most antibacterial agents tested, with its susceptibility to AMK, COL and FEP being 93.5%, 98.0% and 70.6%, respectively. However, *Acinetobacter baumannii* was only susceptible to COL (97.1%) and exhibited a high resistance rate to the antibacterial agents tested. For different species of the genus *Klebsiella*, such as Kp, *K. aerogenes* and *K. oxytoca*, there were significant differences in the sensitivity to FEP. Kp shows a sensitivity rate of 58%, while cefepime remained quite effective against the other two species. The susceptibilities of other GNB to 12 common antibacterial agents are listed in Table 2.

**Table 2:**
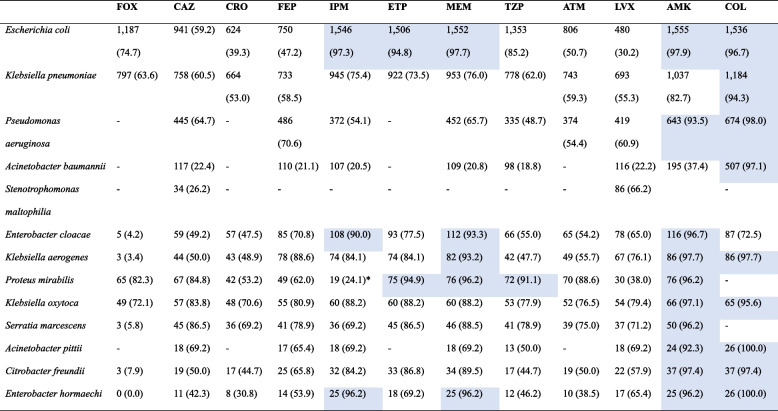
Activity of different antibacterial agents against most common Gram-negative organisms

Susceptibility rates greater than 90%

*Abbreviations*: *AMK *Amikacin, *ATM *Aztreonam, *CAZ *Ceftazidime, *COL *Colistin, *CRO *Ceftriaxone, *ETP *Ertapenem, *FEP *Cefepime, *FOX *Cefoxitin, *IPM *Imipenem, *LVX *Levofloxacin, *MEM *Meropenem, TZP Piperacillin/tazobactam

*Proteus *spp*. tend to have elevated MICs to IPM, caused by intrinsic mechanisms other than by production of carbapenemases. In addition, the Kirby-Bauer method should be used to confirm IPM resistance

- indicates no detection or natural resistance

### Drug susceptibility pattern of different types of drug-resistant GNB isolated from various infection sites

**Fig. 1:**
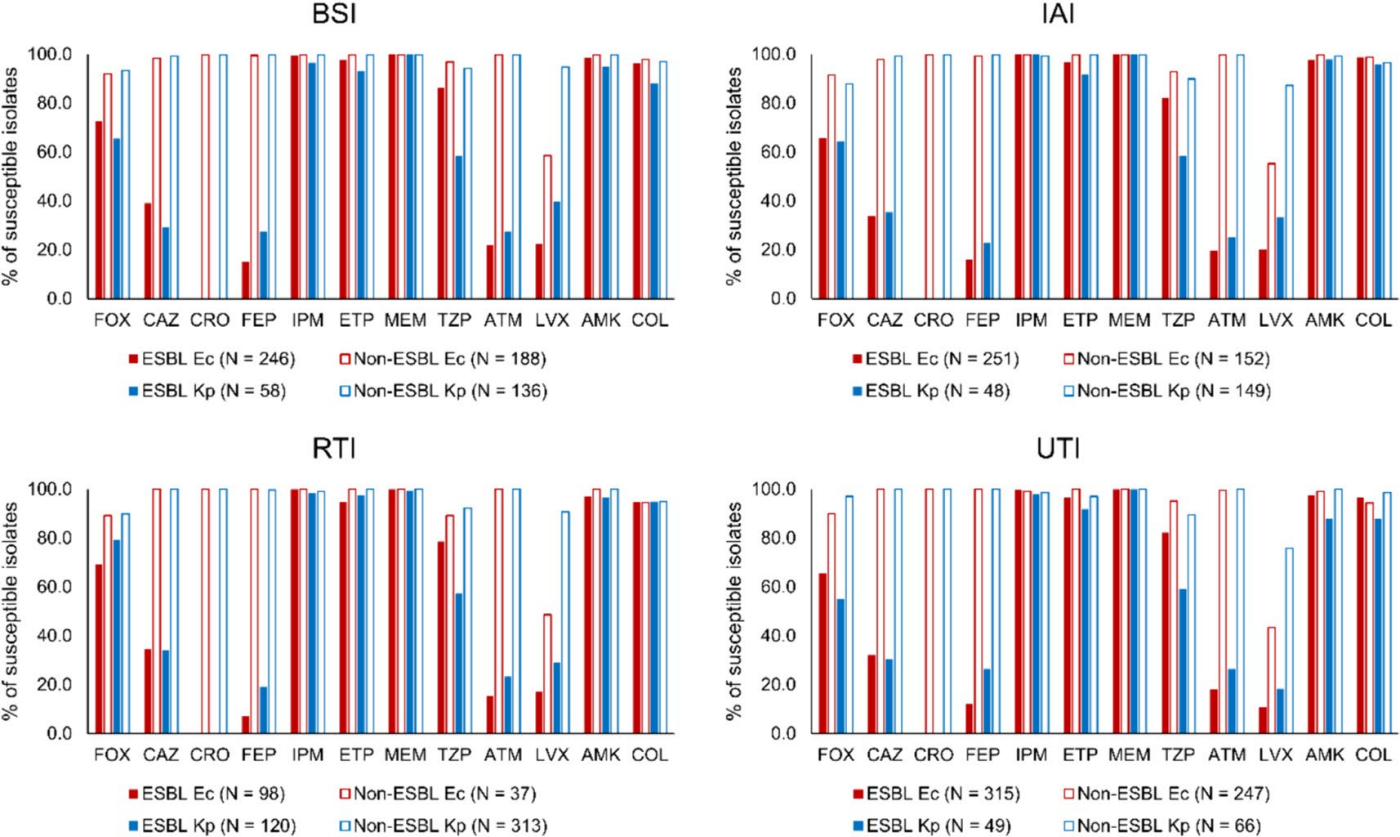
Comparison of the susceptibilities of ESBL and non-ESBL-Ec/Kp in BSI, IAI, RTI and UTI to various antibacterial agents. Abbreviations: AMK, amikacin; ATM, aztreonam; BSI, bloodstream infections; CAZ, ceftazidime; COL, colistin; CRO, ceftriaxone; Ec, *Escherichia coli*; ESBL, extended-spectrum β-lactamases; ETP, ertapenem; FEP, cefepime; FOX, cefoxitin; IAI, intraabdominal infections; IPM, imipenem; Kp, *Klebsiella pneumoniae*; LVX, levofloxacin; MEM, meropenem; RTI, respiratory tract infections; TZP, piperacillin/tazobactam; UTI, urinary tract infections

ESBL producing Ec (ESBL-Ec) and ESBL producing Kp (ESBL-Kp) exhibited distinct antimicrobial susceptibilities despite the type of infection. In BSI, ESBL-Ec and ESBL-Kp still showed high susceptibility (> 96.3% and ≥ 87.9%, respectively) to IPM, ETP, MEM, AMK and COL. In IAI, ESBL-Ec and ESBL-Kp maintained high susceptibility (≥ 96.8% and ≥ 91.7%, respectively) to carbapenems, but were varied in susceptibilities to other antibacterial agents. RTI caused by ESBL-Ec had similar susceptibility rates of ≥ 94.9% to BSI in IPM, ETP, MEM, AMK and COL, while non-ESBL-Ec were 100% consistently susceptible to carbapenems and other antibacterial agents (≥ 89.2%), except for LVX (48.7%). UTI showed ESBL-Ec with susceptibility rates ≥ 96.5% to IPM, ETP, MEM, AMK and COL, and 82.2% to TZP. Non-ESBL-Ec exhibited varied susceptibilities to LVX (43.3%) and consistent susceptibility (≥ 89.9%) to other antibacterial agents. ESBL-Kp had susceptibility rates ≥ 91.8% to carbapenems, 87.8% to AMK and COL, while non-ESBL-Kp exhibited susceptibilities ranging from 89.4% to 100.0%, except for LVX (75.8%). Taken together, regarding ESBL, it is a phenotype that only affects β-lactam antibacterial agents. However, the results showed that for ESBL-producing strains, regardless of the isolation site, their sensitivity to other classes of antibacterial agents was also low, indicating that strains producing ESBL enzymes are more likely to carry resistance genes against other antibacterial agents. Among ESBL-producing strains, 80% of Ec and Kp were multidrug-resistant. In addition, although non-ESBL-producing strains showed high sensitivity to β-lactam antibacterial agents, their sensitivity to LVX was low. Especially for non-ESBL-Ec, the overall sensitivity rate to LVX fluctuated around 50%, implying that during empirical treatment, clinicians must be aware that there is at least a 50% chance of treatment failure with LVX for Ec or Kp (Fig. 1 and Table S1).

### Rates of CRE, CR-Pa and DTR-Pa infections at different sites

Next, we analyzed the detection rates of CRE, CR-Pa, and DTR-Pa at different infection sites. The detection rates of CRE ranged between 10.3% and 18.9%, which were lower than the detection rates of CR-Pa (16.1% – 38.0%). Compared with other species of CRE, CR-Kp in BSI, UTI and RTI accounted for the highest proportion of CRE (24.7% – 35.8%), but the proportions of CR-*Klebsiella aerogenes*, CR-*Enterobacter* *cloacae *and CR-Kp in IAI were 23.8%, 19.4% and 16.2%, respectively. The detection rates of CR-Pa and DTR-Pa varied, with the highest observed in RTI (38.0% and 13.3%) and the lowest in UTI (16.1% and 7.1%) (Table 3).

**Table 3:** Distribution of CRE and CR-Pa in BSI, IAI, RTI and UTI

Microorganisms	Total	BSI	UTI	RTI	IAI
**CRE**	**456 (13.1)**	**102 (12.0)**	**90 (10.3)**	**178 (18.9)**	**86 (10.4)**
CR-Ec	55 (3.5)	12 (2.7)	12 (2.1)	11 (7.5)	20 (4.7)
CR-Kp	317 (25.3)	74 (27.6)	63 (35.8)	142 (24.7)	38 (16.2)
CR-*Enterobacter cloacae*	17 (14.2)	4 (12.5)	4 (23.5)	2 (5.7)	7 (19.4)
CR-*Klebsiella aerogenes*	9 (10.2)	0 (0.0)	0 (0.0)	4 (8.9)	5 (23.8)
CR-*Proteus mirabilis*	4 (5.1)	0 (0.0)	1 (2.7)	2 (11.8)	1 (5.9)
CR-*Klebsiella oxytoca*	8 (11.8)	4 (18.2)	1 (11.1)	3 (14.3)	0 (0.0)
CR-*Serratia marcescens*	8 (15.4)	0 (0.0)	0 (0.0)	8 (25.8)	0 (0.0)
CR-*Citrobacter freundii*	4 (10.5)	1 (14.3)	3 (23.1)	0 (0.0)	0 (0.0)
Others	34 (NA)	7 (NA)	6 (NA)	6 (NA)	15 (NA)
**CR-Pa**	**232 (33.7)**	**14 (22.2)**	**9 (16.1)**	**189 (38.0)**	**20 (27.8)**
**DTR-Pa**	**84 (12.2)**	**6 (9.5)**	**4 (7.1)**	**66 (13.3)**	**8 (11.1)**

Data are presented as numbers (percentages)

Abbreviations: *BSI *Bloodstream infections, *CR *Carbapenem-resistant, *CRE *Carbapenem-resistant Enterobacterales, *DTR *Difficult-to-treat resistance, *Ec* *Escherichia coli*, *Kp* *Klebsiella pneumoniae*, *NA *Not applicable, *IAI *Intraabdominal infections, *Pa* *Pseudomonas aeruginosa*, *RTI *Respiratory tract infections, *UTI *Urinary tract infections

Based on the susceptibility rates of CRE to conventional antibacterial agents, only COL had a susceptibility rate of 88.0% and AMK showed a susceptibility rate of 50.0%, while the susceptibility rates to other commonly used antibacterial agents were ≤ 15.3%. The susceptibility rate of CR-Ec strains to AMK was 80.0%, for CR-Kp against AMK 36.3%, and for CR-Pa against AMK 82.3%, indicating that the CR strains collected in the present study achieved very high resistances to other antibacterial agents. AMK is a powerful antibacterial agent, that has antibacterial potential against CR-Ec and CR-Pa. Moreover, it was found that the susceptibility of CR-Ec isolated from BSI, IAI, RTI and UTI sites to AMK varied (BSI: 83.3%, IAI: 90.0%, RTI: 72.7%, UTI: 66.7%), and the susceptibility of CR-Kp from any infection site to AMK was ≤ 38.0%. Comparably, CR-Pa isolated from BSI, IAI, RTI and UTI exhibited high susceptibility to AMK (100.0%, 80.0%, 82.0% and 66.7%, respectively). Of concern were the resistance patterns of DTR-Pa isolates, which showed high resistance rates for all included antibacterial agents except for AMK, though the DTR-Pa incidence has only been high in RTIs (Table S2).

## Discussion

Given its epidemiological importance, knowledge of the susceptibility of GNB to antibacterial agents is crucial for initiating empirical therapy, as well as for efforts to control the spread of ESBL and CRE [18–20]. Overall comparison of CRE, CR-Ec, CR-Kp, and ESBL-E, ESBL-Ec and ESBL-Kp isolate detections were fairly constant in China during the years 2016 to 2020 (Table S3) being lowest for CR-Ec (3.3%-8.0%), highest for ESBL-Ec (51.7%-59.8%), and intermediate for ESBL-Kp (21.6%-28.3%) and CR-Kp (24.3%-29.7% with the exception of 2020 at 11.9%).

For ESBL-E strains, IPM and ETP as well as AMK were the only antibacterial agent with high susceptibilities throughout the years 2016-2020 without essential changes, while COL showed a similar tendency though it is not commonly used as an antibacterial agent for medication in Chinese hospitals due to its beside effects. Otherwise TZP susceptibilities were around 70%. For CRE isolates, only COL susceptibilities were high throughout the years 2016-2020, with constantly low susceptibility rates for all other antibacterial agents, with 60% AMK susceptibility being the highest without significant annual changes (Fig. S1).

The present study identified Ec as the most prevalent GNB infectious species, followed by Kp, Pa and *A. baumannii*. In 2019 and 2020, Ec and Kp exhibited high susceptibilities to carbapenems, with rates exceeding 94.8% and 73.5%, respectively. ESBL-Ec and ESBL-Kp maintained carbapenems susceptibility > 90% for various infections but demonstrated reduced susceptibility to alternative antibacterial agents. Detection rates of CRE varied across infection sites, ranging from 10.3% (UTI) to 18.9% (RTI). CR-Pa displayed diverse detection rates, with the highest in RTI (38.0%) and the lowest in UTI (16.1%).

However, Kp did exhibit a decrease in the activity of ETP in IAI from 96.7% between 2009 and 2015 to 84.2% between 2016 and 2017 [13]. In a recent publication, even though decreases of ETP susceptibility of Enterobacterales isolated from IAI and UTI were observed in most regions of the world, the susceptibility remained > 90% in regions other than Asia [21]. Our present study has revealed a similar decrease in the susceptibility of carbapenems against Ec (94.8%) and Kp (73.6%), findings similar to previous studies.

Since there has been a continuous increase in the rate of occurrence of Enterobacterales with ESBL globally, especially in Asia [22, 23], we further studied the AMR of various ESBL-E and found that ESBL-Ec isolates from BSI, IAI, RTI and UTI possessed high susceptibility to carbapenems, moderate susceptibility to TZP and low susceptibility to LVX, which are the major new findings in the present study and in agreement with previous studies which have demonstrated that IPM, ETP and AMK were the most active antibacterial agents tested against Ec for both IAI (> 97%) and UTI (> 99%), and that there was evidence of loss of activity over the years 2009-2015 [21].

In comparison, ESBL-Kp had a lower susceptibility to carbapenems than ESBL-Ec. Carbapenems are the treatment of choice for infections caused by ESBL-producing bacteria, and several studies have already reported the predominance of ESBL-Kp isolated from hospitals and emphasized its commonness in IAI and UTI [24].

Regardless of the origin of isolates, our data are in line with the findings of previous studies [25–27]. The emergence of highly transmissible CR isolates has narrowed the therapeutic choices, therefore continuous surveillance of changes in Kp in different hospital settings should be a critical determinant of the choice of antibacterial agent(s) for treatment.

The present study also analyzed the detection rates of CRE and CR-Pa infections at different sites, and found that the detection rates of CRE varied with the site and ranged between 10.3% and 18.9%, which are values lower than those reported in previous studies [28, 29]. Compared with other CRE strains, CR-Kp in BSI, UTI and RTI accounted for the highest proportion of CRE in their respective organs (24.7% – 35.8%), suggesting that CR-Kp is a more severe issue in Chinese hospitals. Furthermore, regardless of the variation in susceptibility of CR-Ec, CR-Kp and CR-Pa obtained from BSI, IAI, RTI and UTI infection sites to AMK, only this antibacterial agent had a high susceptibility against CR isolates, while the susceptibility rate to other commonly used antibacterial agents was ≤ 45.0%. These results indicate that the CR strains collected in the present study reached a very high resistance to other antibacterial drugs. AMK causes several toxic side effects, along with other medicinal properties and a narrow spectrum, but it is rarely used clinically nowadays [30]. The main risk factors for CRE infection were exposure to healthcare, including admission to an ICU, medical devices, invasive procedures and abuse of antibacterial agents [31]. Several classes of antibacterial agents were associated with CRE isolation or infection, including carbapenems, cephalosporins, fluoroquinolones and vancomycin [32–34].

A limitation of the present study was that it provided data on bacterial resistance but did not correlate these findings with clinical outcomes, such as treatment success or failure rates, which limits its practical clinical applicability.

In conclusion, the present study confirmed that carbapenems exhibited good *in vitro *activity against ESBL-E isolates recovered from BSI, IAI, UTI and RTI in China, compared to broad spectrum cephalosporins, and an association of penicillins with β-lactamase inhibitors. Although CR isolates still had a low incidence in Chinese hospitals, its treatment has been limited due to its resistance to a wide range of antibacterial agents. Taken the results overall, it is clear that continuous surveillance should be maintained at local and worldwide levels to facilitate the making of clinical decisions on empirical infection treatment and to support efforts to control infections.

## Supplementary Information

Additional file 1: Table S1 Comparison of different antibacterial agents against ESBL and non-ESBL-Ec/Kp isolated from BSI, IAI, RTI and UTI. Table S2 Susceptibility patterns of CRE, CR-Pa and DTR-Pa to different antibacterial agents. Table S3 Isolation (detection) rate of CRE and ESBL-E strains from 2016 to 2020. Fig. S1 *In vitro* susceptibility of CRE and ESBL-E from 2016 to 2020.

## Data Availability

The SMART database is not public and is only accessible to SMART investigators, but the data that support the findings of this study are directly available from MSD China or from the corresponding author Yunsong Yu upon reasonable request and with permission of MSD China.

## Abbreviations

AMK: Amikacin
AMR: Antimicrobial resistance
ATM: Aztreonam
BPPL: Bacterial Priority Pathogens List
BSI: Bloodstream infections
CAZ: Ceftazidime
CLSI: Clinical and Laboratory Standards Institute
CR: Carbapenem-resistant
CRO: Ceftriaxone
DTR: Difficult-to-treat resistance
Ec: *Escherichia coli*
ESBL: Extended-spectrum β-lactamases
ESBL-E: Extended-spectrum β-lactamases-producing Enterobacterales
ESBL-Ec: Extended-spectrum β-lactamases-producing *Escherichia coli*
ESBL-Kp: Extended-spectrum β-lactamases-producing *Klebsiella pneumoniae*
ETP: Ertapenem
EUCAST: European Committee on Antimicrobial Susceptibility Testing
FEP: Cefepime
FOX: Cefoxitin
GLASS: Global Antimicrobial Resistance and Use Surveillance System
GNB: Gram-negative bacteria
IAI: Intraabdominal infections
IPM: Imipenem
Kp: *Klebsiella pneumoniae*
LVX: Levofloxacin
MEM: Meropenem
MICs: Minimum inhibitory concentrations
Pa: *Pseudomonas aeruginosa*
RTI: Respiratory tract infections
SMART: Study for Monitoring Antimicrobial Resistance Trends
TZP: Piperacillin/tazobactam
UTI: Urinary tract infections
WHO: World Health Organization
