# Supplementary material for: Susceptibility of various Gram-negative bacteria to antibacterial agents: SMART in China 2019–2020
Source: BMC Microbiol. 2024 Dec 19;24:524. doi: 10.1186/s12866-024-03526-8 (PMC11656906; doi:10.1186/s12866-024-03526-8)
Supplement: Supplementary file 1 — Additional file 1: Table S1 Comparison of different antibacterial agents against ESBL and non-ESBL-Ec/Kp isolated from BSI, IAI, RTI and UTI. Table S2 Susceptibility patterns of CRE, CR-Pa and DTR-Pa to different antibacterial agents. Table S3 Isolation (detection) rate of CRE and ESBL-E strains from 2016 to 2020. Fig. S1 In vitro susceptibility of CRE and ESBL-E from 2016 to 2020. [file 12866_2024_3526_MOESM1_ESM.docx]

**Table S1** Comparison of different antibacterial agents against ESBL and non-ESBL-Ec/Kp isolated from BSI, IAI, RTI and UTI

|  | BSI | | | | IAI | | | | RTI | | | | UTI | | | |
| --- | --- | --- | --- | --- | --- | --- | --- | --- | --- | --- | --- | --- | --- | --- | --- | --- |
|  | ESBL Ec | Non-ESBL Ec | ESBL Kp | Non-ESBL Kp | ESBL Ec | Non-ESBL Ec | ESBL Kp | Non-ESBL Kp | ESBL Ec | Non-ESBL Ec | ESBL Kp | Non-ESBL Kp | ESBL Ec | Non-ESBL Ec | ESBL Kp | Non-ESBL Kp |
| N | 246 | 188 | 58 | 136 | 251 | 152 | 48 | 149 | 98 | 37 | 120 | 313 | 315 | 247 | 49 | 66 |
| FOX | 72.8 | 92.0 | 65.5 | 93.4 | 65.7 | 91.5 | 64.6 | 87.9 | 69.4 | 89.2 | 79.2 | 89.8 | 65.7 | 89.9 | 55.1 | 97.0 |
| CAZ | 39.0 | 98.4 | 29.3 | 99.3 | 33.9 | 98.0 | 35.4 | 99.3 | 34.7 | 100.0 | 34.2 | 100.0 | 32.1 | 100.0 | 30.6 | 100.0 |
| CRO | 0.0 | 100.0 | 0.0 | 100.0 | 0.0 | 100.0 | 0.0 | 100.0 | 0.0 | 100.0 | 0.0 | 100.0 | 0.0 | 100.0 | 0.0 | 100.0 |
| FEP | 15.0 | 99.5 | 27.6 | 100.0 | 15.9 | 99.3 | 22.9 | 100.0 | 7.1 | 100.0 | 19.2 | 99.7 | 12.1 | 100.0 | 26.5 | 100.0 |
| IPM | 99.6 | 100.0 | 96.6 | 100.0 | 100.0 | 100.0 | 100.0 | 99.3 | 100.0 | 100.0 | 98.3 | 99.0 | 99.7 | 99.2 | 98.0 | 98.5 |
| ETP | 97.6 | 100.0 | 93.1 | 100.0 | 96.8 | 100.0 | 91.7 | 100.0 | 94.9 | 100.0 | 97.5 | 100.0 | 96.5 | 100.0 | 91.8 | 97.0 |
| MEM | 100.0 | 100.0 | 100.0 | 100.0 | 100.0 | 100.0 | 100.0 | 100.0 | 100.0 | 100.0 | 99.2 | 100.0 | 100.0 | 100.0 | 100.0 | 100.0 |
| TZP | 86.2 | 96.8 | 58.6 | 94.1 | 82.1 | 92.8 | 58.3 | 89.9 | 78.6 | 89.2 | 57.5 | 92.3 | 82.2 | 95.1 | 59.2 | 89.4 |
| ATM | 22.0 | 100.0 | 27.6 | 100.0 | 19.5 | 100.0 | 25.0 | 100.0 | 15.3 | 100.0 | 23.3 | 100.0 | 18.1 | 99.6 | 26.5 | 100.0 |
| LVX | 22.4 | 58.5 | 39.7 | 94.9 | 20.3 | 55.3 | 33.3 | 87.3 | 17.4 | 48.7 | 29.2 | 90.7 | 10.8 | 43.3 | 18.4 | 75.8 |
| AMK | 98.4 | 100.0 | 94.8 | 100.0 | 97.6 | 100.0 | 97.9 | 99.3 | 96.9 | 100.0 | 96.7 | 100.0 | 97.5 | 99.2 | 87.8 | 100.0 |
| COL | 96.3 | 97.9 | 87.9 | 97.1 | 98.8 | 98.7 | 95.8 | 96.6 | 94.9 | 94.6 | 95.0 | 94.9 | 96.5 | 94.3 | 87.8 | 98.5 |

AMK, amikacin; ATM, aztreonam; BSI, bloodstream infections; CAZ, ceftazidime; COL, colistin; CRO, ceftriaxone; Ec, *Escherichia coli*; ESBL, extended-spectrum β-lactamases; ETP, ertapenem; FEP, cefepime; FOX, cefoxitin; IAI, intraabdominal infections; IPM, imipenem; Kp, *Klebsiella pneumoniae*; LVX, levofloxacin; MEM, meropenem; RTI, respiratory tract infections; TZP, piperacillin/tazobactam; UTI, urinary tract infections.

**Table S2** Susceptibility patterns of CRE, CR-Pa and DTR-Pa to different antibacterial agents

| Infection source/phenotype/species | Agent | MIC range | MIC_50_ | MIC_90_ | S (%) | I (%) | R (%) |
| --- | --- | --- | --- | --- | --- | --- | --- |
| **Total** |  |  |  |  |  |  |  |
| **CRE# (N = 450)** | FOX | ≤ 4 to > 16 | > 16 | > 16 | 1.6 | 4.0 | 94.4 |
|  | CAZ | ≤ 1 to > 16 | > 16 | > 16 | 7.1 | 0.9 | 92.0 |
|  | CRO | ≤ 1 to > 8 | > 8 | > 8 | 2.9 | 0.2 | 96.9 |
|  | FEP | ≤ 1 to > 16 | > 16 | > 16 | 8.4 | 2.7 | 88.9 |
|  | IPM | ≤ 0.12 to > 16 | > 16 | > 16 | 11.3 | 1.8 | 86.9 |
|  | ETP | ≤ 0.12 to > 4 | > 4 | > 4 | 3.1 | 0.0 | 96.9 |
|  | MEM | ≤ 0.12 to > 16 | > 16 | > 16 | 15.3 | 2.2 | 82.4 |
|  | TZP | ≤ 4 to > 64 | > 64 | > 64 | 6.9 | 0.9 | 92.2 |
|  | ATM | ≤ 1 to > 8 | > 8 | > 8 | 8.4 | 1.3 | 90.2 |
|  | LVX | ≤ 0.5 to > 4 | > 4 | > 4 | 10.7 | 3.6 | 85.8 |
|  | AMK | ≤ 8 to > 32 | 16 | > 32 | 50.0 | 0.4 | 49.6 |
|  | COL | ≤ 1 to > 4 | ≤ 1 | > 4 | 88.0 | NA | 12.0 |
| CR-Ec (N = 55) | FOX | 8 to > 16 | > 16 | > 16 | 1.8 | 9.1 | 89.1 |
|  | CAZ | ≤ 1 to > 16 | > 16 | > 16 | 12.7 | 0.0 | 87.3 |
|  | CRO | > 4 to > 8 | > 8 | > 8 | 0.0 | 0.0 | 100.0 |
|  | FEP | ≤ 1 to > 16 | > 16 | > 16 | 10.9 | 1.8 | 87.3 |
|  | IPM | ≤ 0.12 to > 16 | 16 | > 16 | 29.1 | 0.0 | 70.9 |
|  | ETP | ≤ 0.12 to > 4 | > 4 | > 4 | 3.6 | 0.0 | 96.4 |
|  | MEM | ≤ 0.12 to > 16 | > 16 | > 16 | 32.7 | 3.6 | 63.6 |
|  | TZP | ≤ 4 to > 64 | > 64 | > 64 | 14.6 | 0.0 | 85.5 |
|  | ATM | ≤ 1 to > 8 | > 8 | > 8 | 14.6 | 9.1 | 76.4 |
|  | LVX | ≤ 0.5 to > 4 | > 4 | > 4 | 7.3 | 1.8 | 90.9 |
|  | AMK | ≤ 8 to > 32 | ≤ 8 | > 32 | 80.0 | 0.0 | 20.0 |
|  | COL | ≤ 1 to > 4 | ≤ 1 | 2 | 94.6 | NA | 5.5 |
| CR-Kp (N = 317) | FOX | ≤ 4 to > 16 | > 16 | > 16 | 1.3 | 1.6 | 97.2 |
|  | CAZ | ≤ 1 to > 16 | > 16 | > 16 | 2.5 | 1.0 | 96.5 |
|  | CRO | ≤ 1 to > 8 | > 8 | > 8 | 0.6 | 0.3 | 99.1 |
|  | FEP | ≤ 1 to > 16 | > 16 | > 16 | 2.8 | 1.0 | 96.2 |
|  | IPM | 0.25 to > 16 | > 16 | > 16 | 5.4 | 1.6 | 93.1 |
|  | ETP | 2 to > 4 | > 4 | > 4 | 0.0 | 0.0 | 100.0 |
|  | MEM | ≤ 0.12 to > 16 | > 16 | > 16 | 5.4 | 1.9 | 92.7 |
|  | TZP | ≤ 4 to > 64 | > 64 | > 64 | 2.5 | 0.3 | 97.2 |
|  | ATM | ≤ 1 to > 8 | > 8 | > 8 | 3.8 | 0.3 | 95.9 |
|  | LVX | ≤ 0.5 to > 4 | > 4 | > 4 | 5.7 | 1.6 | 92.7 |
|  | AMK | ≤ 8 to > 32 | > 32 | > 32 | 36.3 | 0.3 | 63.4 |
|  | COL | ≤ 1 to > 4 | ≤1 | 2 | 92.1 | NA | 7.9 |
| **CR-Pa** (N = 232) | CAZ | ≤ 1 to > 32 | 32 | > 32 | 42.2 | 6.0 | 51.7 |
|  | FEP | ≤ 1 to > 32 | 16 | > 32 | 44.4 | 15.5 | 40.1 |
|  | IPM | 0.5 to > 32 | 16 | > 32 | 0.9 | 2.2 | 97.0 |
|  | MEM | ≤ 0.25 to > 32 | 8 | > 32 | 9.1 | 17.7 | 73.3 |
|  | TZP | ≤ 4 to > 64 | > 64 | > 64 | 22.4 | 6.0 | 71.6 |
|  | ATM | ≤ 1 to > 16 | > 16 | > 16 | 23.7 | 17.2 | 59.1 |
|  | LVX | ≤ 0.5 to > 4 | 4 | > 4 | 28.9 | 19.0 | 52.2 |
|  | AMK | ≤ 4 to > 32 | ≤ 4 | > 32 | 82.3 | 2.6 | 15.1 |
|  | COL | ≤ 1 to > 4 | 2 | 4 | 96.9 | NA | 3.1 |
| **DTR-Pa** (N = 84) | CAZ | 16 to > 32 | > 32 | > 32 | 0.0 | 2.4 | 97.6 |
|  | FEP | 16 to > 32 | > 32 | > 32 | 0.0 | 20.2 | 79.8 |
|  | IPM | 4 to > 32 | 32 | > 32 | 0.0 | 2.4 | 97.6 |
|  | MEM | 4 to > 32 | 32 | > 32 | 0.0 | 4.8 | 95.2 |
|  | TZP | 32 to > 64 | > 64 | > 64 | 0.0 | 1.2 | 98.8 |
|  | ATM | 16 to > 16 | > 16 | > 16 | 0.0 | 6.0 | 94.1 |
|  | LVX | 2 to > 4 | > 4 | > 4 | 0.0 | 15.5 | 84.5 |
|  | AMK | ≤ 4 to > 32 | 8 | > 32 | 66.7 | 2.4 | 31.0 |
| **BSI** |  |  |  |  |  |  |  |
| **CRE#** (N = 101) | FOX | 8 to > 16 | > 16 | > 16 | 2.0 | 3.0 | 95.1 |
|  | CAZ | ≤ 1 to > 16 | > 16 | > 16 | 5.0 | 0.0 | 95.1 |
|  | CRO | ≤ 1 to > 8 | > 8 | > 8 | 2.0 | 0.0 | 98.0 |
|  | FEP | ≤ 1 to > 16 | > 16 | > 16 | 7.9 | 0.0 | 92.1 |
|  | IPM | 0.25 to > 16 | > 16 | > 16 | 11.9 | 1.0 | 87.1 |
|  | ETP | ≤ 0.12 to > 4 | > 4 | > 4 | 3.0 | 0.0 | 97.0 |
|  | MEM | ≤ 0.12 to > 16 | > 16 | > 16 | 15.8 | 2.0 | 82.2 |
|  | TZP | ≤ 4 to > 64 | > 64 | > 64 | 5.9 | 2.0 | 92.1 |
|  | ATM | ≤ 1 to > 8 | > 8 | > 8 | 8.9 | 1.0 | 90.1 |
|  | LVX | ≤ 0.5 to > 4 | > 4 | > 4 | 11.9 | 4.0 | 84.2 |
|  | AMK | ≤ 8 to > 32 | > 32 | > 32 | 49.5 | 0.0 | 50.5 |
|  | COL | ≤ 1 to > 4 | ≤ 1 | 2 | 91.1 | NA | 8.9 |
| CR-Ec (N = 12) | FOX | 8 to > 16 | > 16 | > 16 | 8.3 | 8.3 | 83.3 |
|  | CAZ | ≤ 1 to > 16 | > 16 | > 16 | 16.7 | 0.0 | 83.3 |
|  | CRO | > 4 to > 8 | > 8 | > 8 | 0.0 | 0.0 | 100.0 |
|  | FEP | ≤ 1 to > 16 | > 16 | > 16 | 16.7 | 0.0 | 83.3 |
|  | IPM | 0.25 to > 16 | 16 | > 16 | 25.0 | 0.0 | 75.0 |
|  | ETP | ≤ 0.12 to > 4 | > 4 | > 4 | 16.7 | 0.0 | 83.3 |
|  | MEM | ≤ 0.12 to > 16 | 8 | > 16 | 33.3 | 8.3 | 58.3 |
|  | TZP | ≤ 4 to > 64 | > 64 | > 64 | 25.0 | 0.0 | 75.0 |
|  | ATM | ≤ 1 to > 8 | > 8 | > 8 | 16.7 | 8.3 | 75.0 |
|  | LVX | ≤ 0.5 to > 4 | > 4 | > 4 | 8.3 | 0.0 | 91.7 |
|  | AMK | ≤ 8 to > 32 | ≤ 8 | > 32 | 83.3 | 0.0 | 16.7 |
|  | COL | ≤ 1 to 2 | ≤ 1 | 2 | 100.0 | NA | 0.0 |
| CR-Kp (N = 74) | FOX | 8 to > 16 | > 16 | > 16 | 1.4 | 1.4 | 97.3 |
|  | CAZ | ≤ 1 to > 16 | > 16 | > 16 | 1.4 | 0.0 | 98.7 |
|  | CRO | 4 to > 8 | > 8 | > 8 | 0.0 | 0.0 | 100.0 |
|  | FEP | ≤ 1 to > 16 | > 16 | > 16 | 2.7 | 0.0 | 97.3 |
|  | IPM | 0.25 to > 16 | > 16 | > 16 | 6.8 | 0.0 | 93.2 |
|  | ETP | 2 to > 4 | > 4 | > 4 | 0.0 | 0.0 | 100.0 |
|  | MEM | ≤ 0.12 to > 16 | > 16 | > 16 | 8.1 | 1.4 | 90.5 |
|  | TZP | ≤ 4 to > 64 | > 64 | > 64 | 2.7 | 0.0 | 97.3 |
|  | ATM | ≤ 1 to > 8 | > 8 | > 8 | 5.4 | 0.0 | 94.6 |
|  | LVX | ≤ 0.5 to > 4 | > 4 | > 4 | 8.1 | 0.0 | 91.9 |
|  | AMK | ≤ 8 to > 32 | > 32 | > 32 | 36.5 | 0.0 | 63.5 |
|  | COL | ≤ 1 to > 4 | ≤ 1 | 2 | 91.9 | NA | 8.1 |
| **CR-Pa** (N = 14) | CAZ | 4 to > 32 | 16 | > 32 | 42.9 | 14.3 | 42.9 |
|  | FEP | 4 to > 32 | 16 | > 32 | 42.9 | 14.3 | 42.9 |
|  | IPM | 4 to > 32 | 16 | > 32 | 0.0 | 14.3 | 85.7 |
|  | MEM | 2 to > 32 | 16 | > 32 | 7.1 | 0.0 | 92.9 |
|  | TZP | 8 to > 64 | > 64 | > 64 | 7.1 | 7.1 | 85.7 |
|  | ATM | 4 to > 16 | > 16 | > 16 | 14.3 | 14.3 | 71.4 |
|  | LVX | ≤ 0.5 to > 4 | 2 | > 4 | 28.6 | 21.4 | 50.0 |
|  | AMK | ≤ 4 to 16 | ≤ 4 | 16 | 100.0 | 0.0 | 0.0 |
|  | COL | ≤ 1 to 4 | 2 | 4 | 100.0 | NA | 0.0 |
| **DTR-Pa** (N = 6) | CAZ | 16 to > 32 | > 32 | > 32 | 0.0 | 16.7 | 83.3 |
|  | FEP | 16 to > 32 | > 32 | > 32 | 0.0 | 16.7 | 83.3 |
|  | IPM | 8 to > 32 | > 32 | > 32 | 0.0 | 0.0 | 100.0 |
|  | MEM | 16 to > 32 | 32 | > 32 | 0.0 | 0.0 | 100.0 |
|  | TZP | 32 to > 64 | > 64 | > 64 | 0.0 | 16.7 | 83.3 |
|  | ATM | > 16 to > 16 | > 16 | > 16 | 0.0 | 0.0 | 100.0 |
|  | LVX | ≤ 4 to 16 | ≤ 4 | 16 | 0.0 | 16.7 | 83.3 |
|  | AMK | 2 to > 4 | > 4 | > 4 | 100.0 | 0.0 | 0.0 |
| **IAI** |  |  |  |  |  |  |  |
| **CRE#** (N = 84) | FOX | 8 to > 16 | > 16 | > 16 | 1.2 | 7.1 | 91.7 |
|  | CAZ | ≤ 1 to > 16 | > 16 | > 16 | 9.5 | 2.4 | 88.1 |
|  | CRO | ≤ 1 to > 8 | > 8 | > 8 | 7.1 | 0.0 | 92.9 |
|  | FEP | ≤ 1 to > 16 | > 16 | > 16 | 15.5 | 9.5 | 75.0 |
|  | IPM | ≤ 0.12 to > 16 | > 16 | > 16 | 26.2 | 1.2 | 72.6 |
|  | ETP | ≤ 0.12 to > 4 | > 4 | > 4 | 6.0 | 0.0 | 94.1 |
|  | MEM | ≤ 0.12 to > 16 | > 16 | > 16 | 29.8 | 2.4 | 67.9 |
|  | TZP | ≤ 4 to > 64 | > 64 | > 64 | 13.1 | 1.2 | 85.7 |
|  | ATM | ≤ 1 to > 8 | > 8 | > 8 | 15.5 | 1.2 | 83.3 |
|  | LVX | ≤ 0.5 to > 4 | > 4 | > 4 | 19.1 | 3.6 | 77.4 |
|  | AMK | ≤ 8 to > 32 | ≤ 8 | > 32 | 69.1 | 0.0 | 31.0 |
|  | COL | ≤ 1 to > 4 | ≤ 1 | > 4 | 84.5 | NA | 15.5 |
| CR-Ec (N = 20) | FOX | 16 to > 16 | > 16 | > 16 | 0.0 | 15.0 | 85.0 |
|  | CAZ | ≤ 1 to > 16 | > 16 | > 16 | 5.0 | 0.0 | 95.0 |
|  | CRO | > 4 to > 8 | > 8 | > 8 | 0.0 | 0.0 | 100.0 |
|  | FEP | ≤ 1 to > 16 | > 16 | > 16 | 5.0 | 5.0 | 90.0 |
|  | IPM | ≤ 0.12 to > 16 | 8 | > 16 | 40.0 | 0.0 | 60.0 |
|  | ETP | 2 to > 4 | > 4 | > 4 | 0.0 | 0.0 | 100.0 |
|  | MEM | ≤ 0.12 to > 16 | > 16 | > 16 | 40.0 | 0.0 | 60.0 |
|  | TZP | ≤ 4 to > 64 | > 64 | > 64 | 10.0 | 0.0 | 90.0 |
|  | ATM | ≤ 1 to > 8 | > 8 | > 8 | 20.0 | 5.0 | 75.0 |
|  | LVX | ≤ 0.5 to > 4 | > 4 | > 4 | 5.0 | 5.0 | 90.0 |
|  | AMK | ≤ 8 to > 32 | ≤ 8 | 16 | 90.0 | 0.0 | 10.0 |
|  | COL | ≤ 1 to > 4 | ≤ 1 | 2 | 90.0 | NA | 10.0 |
| CR-Kp (N = 38) | FOX | > 16 to > 16 | > 16 | > 16 | 0.0 | 0.0 | 100.0 |
|  | CAZ | 2 to > 16 | > 16 | > 16 | 2.6 | 2.6 | 94.7 |
|  | CRO | > 4 to > 8 | > 8 | > 8 | 0.0 | 0.0 | 100.0 |
|  | FEP | 2 to > 16 | > 16 | > 16 | 2.6 | 0.0 | 97.4 |
|  | IPM | 0.5 to > 16 | > 16 | > 16 | 7.9 | 2.6 | 89.5 |
|  | ETP | 2 to > 4 | > 4 | > 4 | 0.0 | 0.0 | 100.0 |
|  | MEM | 0.5 to > 16 | > 16 | > 16 | 2.6 | 2.6 | 94.7 |
|  | TZP | 8 to > 64 | > 64 | > 64 | 5.3 | 0.0 | 94.7 |
|  | ATM | ≤ 1 to > 8 | > 8 | > 8 | 5.3 | 0.0 | 94.7 |
|  | LVX | ≤ 0.5 to > 4 | > 4 | > 4 | 5.3 | 2.6 | 92.1 |
|  | AMK | ≤ 8 to > 32 | > 32 | > 32 | 36.8 | 0.0 | 63.2 |
|  | COL | ≤ 1 to > 4 | ≤ 1 | > 4 | 86.8 | NA | 13.2 |
| **CR-Pa** (N = 20) | CAZ | 2 to > 32 | 32 | > 32 | 45.0 | 0.0 | 55.0 |
|  | FEP | 2 to > 32 | 16 | > 32 | 45.0 | 20.0 | 35.0 |
|  | IPM | 8 to > 32 | 16 | > 32 | 0.0 | 0.0 | 100.0 |
|  | MEM | 2 to > 32 | 8 | > 32 | 15.0 | 15.0 | 70.0 |
|  | TZP | ≤ 4 to > 64 | > 64 | > 64 | 25.0 | 5.0 | 70.0 |
|  | ATM | 4 to > 16 | 16 | > 16 | 25.0 | 25.0 | 50.0 |
|  | LVX | ≤ 0.5 to > 4 | 2 | > 4 | 30.0 | 25.0 | 45.0 |
|  | AMK | ≤ 4 to > 32 | ≤ 4 | > 32 | 80.0 | 0.0 | 20.0 |
|  | COL | ≤ 1 to > 4 | 2 | 2 | 95.0 | NA | 5.0 |
| **DTR-Pa** (N = 8) | CAZ | 32 to > 32 | > 32 | > 32 | 0.0 | 0.0 | 100.0 |
|  | FEP | 16 to > 32 | > 32 | > 32 | 0.0 | 25.0 | 75.0 |
|  | IPM | 8 to > 32 | > 32 | > 32 | 0.0 | 0.0 | 100.0 |
|  | MEM | 8 to > 32 | > 32 | > 32 | 0.0 | 0.0 | 100.0 |
|  | TZP | > 64 to > 64 | > 64 | > 64 | 0.0 | 0.0 | 100.0 |
|  | ATM | > 16 to > 16 | > 16 | > 16 | 0.0 | 0.0 | 100.0 |
|  | LVX | 2 to > 4 | > 4 | > 4 | 0.0 | 12.5 | 87.5 |
|  | AMK | ≤ 4 to > 32 | > 32 | > 32 | 50.0 | 0.0 | 50.0 |
| **RTI** |  |  |  |  |  |  |  |
| **CRE#** (N = 176) | FOX | 8 to > 16 | > 16 | > 16 | 1.1 | 2.8 | 96.0 |
|  | CAZ | ≤ 1 to > 16 | > 16 | > 16 | 6.3 | 1.1 | 92.6 |
|  | CRO | ≤ 1 to > 8 | > 8 | > 8 | 0.6 | 0.0 | 99.4 |
|  | FEP | ≤ 1 to > 16 | > 16 | > 16 | 5.1 | 1.7 | 93.2 |
|  | IPM | 0.25 to > 16 | > 16 | > 16 | 3.4 | 1.1 | 95.5 |
|  | ETP | ≤ 0.12 to > 4 | > 4 | > 4 | 0.6 | 0.0 | 99.4 |
|  | MEM | ≤ 0.12 to > 16 | > 16 | > 16 | 6.3 | 1.1 | 92.6 |
|  | TZP | ≤ 4 to > 64 | > 64 | > 64 | 2.3 | 0.0 | 97.7 |
|  | ATM | ≤ 1 to > 8 | > 8 | > 8 | 2.3 | 1.1 | 96.6 |
|  | LVX | ≤ 0.5 to > 4 | > 4 | > 4 | 8.0 | 2.8 | 89.2 |
|  | AMK | ≤ 8 to > 32 | > 32 | > 32 | 43.8 | 0.0 | 56.3 |
|  | COL | ≤ 1 to > 4 | ≤ 1 | 4 | 89.2 | NA | 10.8 |
| CR-Ec (N = 11) | FOX | > 16 to > 16 | > 16 | > 16 | 0.0 | 0.0 | 100.0 |
|  | CAZ | ≤ 1 to > 16 | > 16 | > 16 | 27.3 | 0.0 | 72.7 |
|  | CRO | > 4 to > 8 | > 8 | > 8 | 0.0 | 0.0 | 100.0 |
|  | FEP | 2 to > 16 | > 16 | > 16 | 18.2 | 0.0 | 81.8 |
|  | IPM | 0.5 to > 16 | 16 | > 16 | 9.1 | 0.0 | 90.9 |
|  | ETP | 2 to > 4 | > 2 | > 4 | 0.0 | 0.0 | 100.0 |
|  | MEM | 0.25 to > 16 | > 16 | > 16 | 27.3 | 0.0 | 72.7 |
|  | TZP | 8 to > 64 | > 64 | > 64 | 9.1 | 0.0 | 90.9 |
|  | ATM | ≤ 1 to > 8 | > 8 | > 8 | 18.2 | 18.2 | 63.6 |
|  | LVX | ≤ 0.5 to > 4 | > 4 | > 4 | 18.2 | 0.0 | 81.8 |
|  | AMK | ≤ 8 to > 32 | ≤ 8 | > 32 | 72.7 | 0.0 | 27.3 |
|  | COL | ≤ 1 to > 4 | ≤ 1 | 2 | 90.9 | NA | 9.1 |
| CR-Kp (N = 142) | FOX | 8 to > 16 | > 16 | > 16 | 0.7 | 2.8 | 96.5 |
|  | CAZ | 4 to > 16 | > 16 | > 16 | 1.4 | 1.4 | 97.2 |
|  | CRO | > 4 to > 8 | > 8 | > 8 | 0.0 | 0.0 | 100.0 |
|  | FEP | ≤ 1 to > 16 | > 16 | > 16 | 1.4 | 2.1 | 96.5 |
|  | TZP | ≤ 4 to > 64 | > 64 | > 64 | 0.7 | 0.0 | 99.3 |
|  | IPM | 0.25 to > 16 | > 16 | > 16 | 2.8 | 0.0 | 97.2 |
|  | ETP | 2 to > 4 | > 4 | > 4 | 0.0 | 0.0 | 100.0 |
|  | MEM | ≤ 0.12 to > 16 | > 16 | > 16 | 2.1 | 1.4 | 96.5 |
|  | ATM | > 8 to > 8 | > 8 | > 8 | 0.0 | 0.0 | 100.0 |
|  | LVX | ≤ 0.5 to > 4 | > 4 | > 4 | 4.9 | 1.4 | 93.7 |
|  | AMK | ≤ 8 to > 32 | > 32 | > 32 | 38.0 | 0.0 | 62.0 |
|  | COL | ≤ 1 to > 4 | ≤ 1 | 2 | 95.1 | NA | 4.9 |
| **CR-Pa** (N = 189) | CAZ | ≤ 1 to > 32 | 32 | > 32 | 41.8 | 6.4 | 51.9 |
|  | FEP | ≤ 1 to > 32 | 16 | > 32 | 44.4 | 15.9 | 39.7 |
|  | IPM | 0.5 to > 32 | 16 | > 32 | 1.1 | 1.6 | 97.4 |
|  | MEM | ≤ 0.25 to > 32 | 16 | > 32 | 7.9 | 18.5 | 73.5 |
|  | TZP | ≤ 4 to > 64 | > 64 | > 64 | 22.8 | 6.4 | 70.9 |
|  | ATM | ≤ 1 to > 16 | > 16 | > 16 | 24.9 | 16.4 | 58.7 |
|  | LVX | ≤ 0.5 to > 4 | 4 | > 4 | 29.6 | 18.5 | 51.9 |
|  | AMK | ≤ 4 to > 32 | ≤ 4 | > 32 | 82.0 | 3.2 | 14.8 |
|  | COL | ≤ 1 to > 4 | 2 | 4 | 96.8 | NA | 3.2 |
| **DTR-Pa** (N = 66) | CAZ | 16 to > 32 | > 32 | > 32 | 0.0 | 1.5 | 98.5 |
|  | FEP | 16 to > 32 | 32 | > 32 | 0.0 | 21.2 | 78.8 |
|  | IPM | 4 to > 32 | 16 | > 32 | 0.0 | 3.0 | 97.0 |
|  | MEM | 4 to > 32 | 16 | > 32 | 0.0 | 6.1 | 93.9 |
|  | TZP | 64 to > 64 | > 64 | > 64 | 0.0 | 0.0 | 100.0 |
|  | ATM | 16 to > 16 | > 16 | > 16 | 0.0 | 7.6 | 92.4 |
|  | LVX | 2 to > 4 | > 4 | > 4 | 0.0 | 16.7 | 83.3 |
|  | AMK | ≤ 4 to > 32 | 8 | > 32 | 68.2 | 3.0 | 28.8 |
| **UTI** |  |  |  |  |  |  |  |
| **CRE#** (N = 89) | FOX | ≤ 4 to > 16 | > 16 | > 16 | 2.3 | 4.5 | 93.3 |
|  | CAZ | ≤ 1 to > 16 | > 16 | > 16 | 9.0 | 0.0 | 91.0 |
|  | CRO | ≤ 1 to > 8 | > 8 | > 8 | 4.5 | 1.1 | 94.4 |
|  | FEP | ≤ 1 to > 16 | > 16 | > 16 | 9.0 | 1.1 | 89.9 |
|  | IPM | ≤ 0.12 to > 16 | > 16 | > 16 | 12.4 | 4.5 | 83.2 |
|  | ETP | ≤ 0.12 to > 4 | > 4 | > 4 | 5.6 | 0.0 | 94.4 |
|  | MEM | ≤ 0.12 to > 16 | > 16 | > 16 | 19.1 | 4.5 | 76.4 |
|  | TZP | ≤ 4 to > 64 | > 64 | > 64 | 11.2 | 1.1 | 87.6 |
|  | ATM | ≤ 1 to > 8 | > 8 | > 8 | 13.5 | 2.3 | 84.3 |
|  | LVX | ≤ 0.5 to > 4 | > 4 | > 4 | 6.7 | 4.5 | 88.8 |
|  | AMK | ≤ 8 to > 32 | > 32 | > 32 | 44.9 | 2.3 | 52.8 |
|  | COL | ≤ 1 to > 4 | ≤ 1 | > 4 | 85.4 | NA | 14.6 |
| CR-Ec (N = 12) | FOX | 16 to > 16 | > 16 | > 16 | 0.0 | 8.3 | 91.7 |
|  | CAZ | ≤ 1 to > 16 | > 16 | > 16 | 8.3 | 0.0 | 91.7 |
|  | CRO | > 4 to > 8 | > 8 | > 8 | 0.0 | 0.0 | 100.0 |
|  | FEP | 2 to > 16 | > 16 | > 16 | 8.3 | 0.0 | 91.7 |
|  | IPM | ≤ 0.12 to > 16 | 16 | > 16 | 33.3 | 0.0 | 66.7 |
|  | ETP | 2 to > 4 | > 4 | > 4 | 0.0 | 0.0 | 100.0 |
|  | MEM | ≤ 0.12 to > 16 | 16 | > 16 | 25.0 | 8.3 | 66.7 |
|  | TZP | ≤ 4 to > 64 | > 64 | > 64 | 16.7 | 0.0 | 83.3 |
|  | ATM | 8 to > 8 | > 8 | > 8 | 0.0 | 8.3 | 91.7 |
|  | LVX | 2 to > 4 | > 4 | > 4 | 0.0 | 0.0 | 100.0 |
|  | AMK | ≤ 8 to > 32 | ≤ 8 | > 32 | 66.7 | 0.0 | 33.3 |
|  | COL | ≤ 1 to 2 | ≤ 1 | 2 | 100.0 | NA | 0.0 |
| CR-Kp (N = 63) | FOX | ≤ 4 to > 16 | > 16 | > 16 | 3.2 | 0.0 | 96.8 |
|  | CAZ | ≤ 1 to > 16 | > 16 | > 16 | 6.4 | 0.0 | 93.7 |
|  | CRO | ≤ 1 to > 8 | > 8 | > 8 | 3.2 | 1.6 | 95.2 |
|  | FEP | ≤ 1 to > 16 | > 16 | > 16 | 6.4 | 0.0 | 93.7 |
|  | IPM | 0.25 to > 16 | > 16 | > 16 | 7.9 | 6.4 | 85.7 |
|  | ETP | 2 to > 4 | > 4 | > 4 | 0.0 | 0.0 | 100.0 |
|  | MEM | 0.25 to > 16 | > 16 | > 16 | 11.1 | 3.2 | 85.7 |
|  | TZP | ≤ 4 to > 64 | > 64 | > 64 | 4.8 | 1.6 | 93.7 |
|  | ATM | ≤ 1 to > 8 | > 8 | > 8 | 9.5 | 1.6 | 88.9 |
|  | LVX | ≤ 0.5 to > 4 | > 4 | > 4 | 4.8 | 3.2 | 92.1 |
|  | AMK | ≤ 8 to > 32 | > 32 | > 32 | 31.8 | 1.6 | 66.7 |
|  | COL | ≤ 1 to > 4 | ≤ 1 | 4 | 88.9 | NA | 11.1 |
| **CR-Pa** (N = 9) | CAZ | 2 to > 32 | > 32 | > 32 | 44.4 | 0.0 | 55.6 |
|  | FEP | 2 to > 32 | 32 | > 32 | 44.4 | 0.0 | 55.6 |
|  | IPM | 8 to > 32 | 16 | > 32 | 0.0 | 0.0 | 100.0 |
|  | MEM | 0.5 to > 32 | 4 | > 32 | 22.2 | 33.3 | 44.4 |
|  | TZP | 8 to > 64 | > 64 | > 64 | 33.3 | 0.0 | 66.7 |
|  | ATM | 2 to > 16 | > 16 | > 16 | 11.1 | 22.2 | 66.7 |
|  | LVX | ≤ 0.5 to > 4 | > 4 | > 4 | 11.1 | 11.1 | 77.8 |
|  | AMK | ≤ 4 to > 32 | 8 | > 32 | 66.7 | 0.0 | 33.3 |
|  | COL | ≤ 1 to 2 | 2 | 2 | 100.0 | NA | 0.0 |
| **DTR-Pa** (N = 4) | CAZ | > 32 to > 32 | > 32 | > 32 | 0.0 | 0.0 | 100.0 |
|  | FEP | > 32 to > 32 | > 32 | > 32 | 0.0 | 0.0 | 100.0 |
|  | IPM | > 32 to > 32 | > 32 | > 32 | 0.0 | 0.0 | 100.0 |
|  | MEM | > 32 to > 32 | > 32 | > 32 | 0.0 | 0.0 | 100.0 |
|  | TZP | > 64 to > 64 | > 64 | > 64 | 0.0 | 0.0 | 100.0 |
|  | ATM | > 16 to > 16 | > 16 | > 16 | 0.0 | 0.0 | 100.0 |
|  | LVX | > 4 to > 4 | > 4 | > 4 | 0.0 | 0.0 | 100.0 |
|  | AMK | ≤ 4 to > 32 | > 32 | > 32 | 25.0 | 0.0 | 75.0 |

Note: #*Proteus spp. a*nd *Salmonella spp.* were excluded. NA, not applicable.

Abbreviations: AMK, amikacin; ATM, aztreonam; BSI, bloodstream infections; CAZ, ceftazidime; COL, colistin; CR, carbapenem-resistant; CRE, carbapenem-resistant *Enterobacterales*; CRO, ceftriaxone; DTR, difficult-to-treat resistance; Ec, *Escherichia coli*; ESBL, extended-spectrum β-lactamases; ETP, ertapenem; FEP, cefepime; FOX, cefoxitin; I, intermediate rate; IAI, intraabdominal infections; IPM, imipenem; Kp, *Klebsiella pneumoniae*; LVX, levofloxacin; MEM, meropenem; MIC, minimum inhibition concentration; Pa, *Pseudomonas aeruginosa*; R, resistant rate; RTI, respiratory tract infections; S, susceptible rate; TZP, piperacillin/tazobactam; UTI, urinary tract infections.

**Table S3** Isolation (detection) rate of CRE and ESBL-E strains from 2016 to 2020

|  | **2016** | **2017** | **2018** | **2019** | **2020** |
| --- | --- | --- | --- | --- | --- |
| CRE | 363 (15.3%) | 421 (16.6%) | 389 (13.7%) | 399 (14.5%) | 57 (7.7%) |
| CR-Ec | 97 (8.0%) | 87 (7.1%) | 58 (4.4%) | 40 (3.3%) | 15 (4.0%) |
| CR-Kp | 184 (24.3%) | 237 (29.7%) | 263 (25.5%) | 288 (28.5%) | 29 (11.9%) |
| ESBL-E | 996 (41.9%) | 998 (39.4%) | 1091 (38.3%) | 1064 (38.7%) | 317 (42.7%) |
| ESBL-Ec | 664 (54.9%) | 636 (51.7%) | 709 (53.9%) | 684 (56.5%) | 226 (59.8%) |
| ESBL-Kp | 214 (28.3%) | 198 (24.8%) | 245 (23.8%) | 218 (21.6%) | 57 (23.5%) |

Abbreviations: CR, carbapenem-resistant; E, Enterobacterales; Ec, *Escherichia coli*; ESBL, extended-spectrum β-lactamases; Kp, *Klebsiella pneumoniae*.


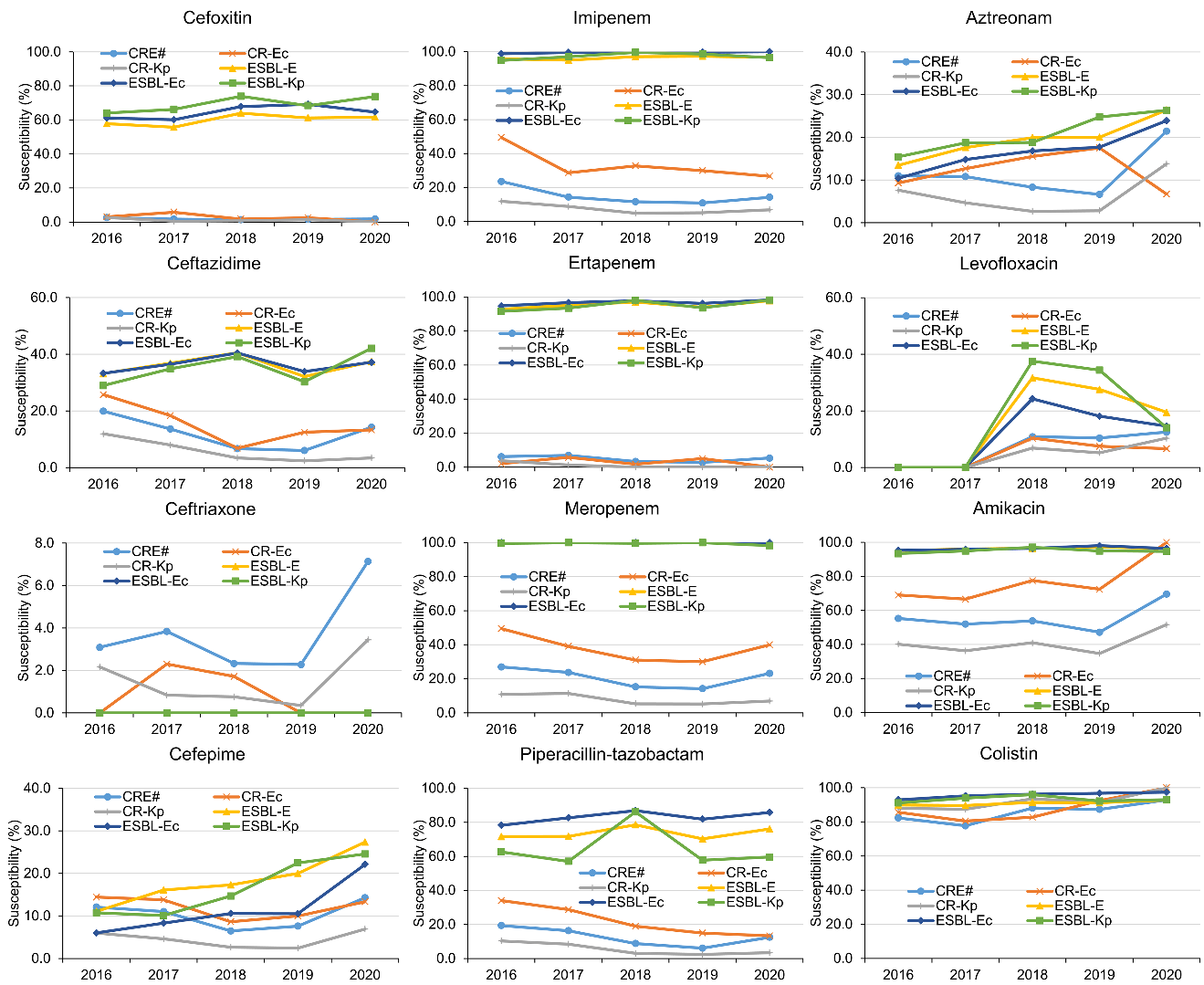


**Fig. S1** *In vitro* susceptibility of CRE and ESBL-E from 2016 to 2020

Note: #*Proteus spp. a*nd *Salmonella spp.* were excluded.

Abbreviations: CR, carbapenem-resistant; CRE, carbapenem-resistant Enterobacterales; Ec, *Escherichia coli*; ESBL, extended-spectrum β-lactamases; Kp, *Klebsiella pneumoniae*.
